# Stakeholder perspectives on the implementation of genetic carrier screening in a changing landscape

**DOI:** 10.1186/s12913-017-2083-9

**Published:** 2017-02-16

**Authors:** Kim C.A. Holtkamp, Evelien M. Vos, Tessel Rigter, Phillis Lakeman, Lidewij Henneman, Martina C. Cornel

**Affiliations:** 1 Department of Clinical Genetics, Section of Community Genetics, Amsterdam Public Health Research Institute, VU University Medical Center, PO Box 7057 (BS7 A-509), 1007 MB Amsterdam, The Netherlands; 2 Department of Clinical Genetics, Academic Medical Center, Amsterdam, The Netherlands

**Keywords:** Carrier screening, Expanded universal carrier screening, Implementation, Stakeholders, Interviews, Barriers, Needs

## Abstract

**Background:**

In most countries, genetic carrier screening is neither offered, nor embedded in mainstream healthcare. Technological developments have triggered a two-fold transition in carrier screening: the expansion from screening one single disorder to many disorders simultaneously, and offering screening universally, regardless of ancestry. This study aims to identify general and population-specific barriers and needs reflected by stakeholders regarding the implementation of carrier screening in a changing landscape.

**Methods:**

Seventeen semi-structured interviews were conducted with Dutch key stakeholders working in the practical and scientific field of carrier screening. The constellation approach was used to categorise barriers and needs into three levels: culture, structure and practice.

**Results:**

Barriers on a cultural level include: undecidedness about the desirability of carrier screening, and a lack of priority of screening in mainstream healthcare. On a structural level barriers included: need for organisational structures in healthcare for embedding carrier screening, need for guidelines, financial structures, practical tools for overcoming challenges during counselling, and a need for training and education of both professionals and the public. A lack of demand for screening by the public, and a need for a division of responsibilities were barriers on a practical level.

**Conclusion:**

The absence of a collective sense of urgency for genetic carrier screening, a lack of organisational structures, and uncertainty or even disagreement about the responsibilities seem to be important barriers in the implementation of carrier screening. Stakeholders therefore suggest that change agents should be formally acknowledged to strategically plan broadening of current initiatives and attune different stakeholders.

**Electronic supplementary material:**

The online version of this article (doi:10.1186/s12913-017-2083-9) contains supplementary material, which is available to authorized users.

## Background

Genetic carrier screening enables couples to find out whether they are both carriers of the same autosomal recessive (AR) disorder, and consequently face a 1-in-4 risk of having an affected child in each pregnancy. The aim of carrier screening is to provide carrier couples with autonomous reproductive choice [1]. Screening is preferably done before pregnancy (preconception) as this provides couples with most reproductive options [2]. These are, for example, refraining from having children, prenatal diagnosis, and preimplantation genetic diagnosis.

In many countries, there has been discussion about the implementation of carrier screening. Currently, screening practices are mostly aimed at high-risk groups, i.e. ancestry-based carrier screening. The Ashkenazi Jewish (AJ) community, for example, has been familiar with carrier screening since the 1970s, starting with screening for Tay-Sachs disease [3]. In England, ancestry-based prenatal haemoglobinopathy (HbP; sickle cell disease, thalassaemia) carrier screening is conducted in low prevalence areas, whereas universal screening, regardless of ancestry, is offered in high prevalence areas [4]. In the United States (US) carrier screening for cystic fibrosis (CF) has been offered preconceptionally or early in pregnancy for over a decade, initially ancestry-based, and since 2011 universally [5].

In most countries, carrier screening has not been implemented on a structural level, and the initiatives that do exist are mostly local or small niches developed from a research perspective [6–9]. For those niches, research has been done to identify possible enabling and inhibiting factors for its implementation and embedding in mainstream healthcare. Challenges or barriers frequently mentioned are: a lack of awareness and knowledge among both the general public and professionals [10–12]; difficulties reaching the target groups, particularly couples planning a pregnancy [10, 11]; professionals being unaware of available guidelines [11, 13]; and a lack of structure in which to embed genetic carrier screening [10, 11].

Technological developments in genetics such as Next Generation Sequencing, and cost reductions [14] have triggered a two-fold transition in carrier screening: the expansion of screening from one single disorder to screening for many disorders simultaneously (‘expanded’), and the transition of an ancestry-based offer towards an offer of screening regardless of ancestry or geographical origin (‘universal’) [15]. Expanded universal carrier screening (EUCS) panels including over 100 disorders, are increasingly available from commercial laboratories in North-America, Australia and Europe [1, 16], and are increasingly being offered to the public [14]. A frequently mentioned advantage of EUCS is that it will reduce stigmatisation and increase equity [1, 17]. However, these advantages may not be as evident as stated. In 2014 and 2015, we conducted an interview study among key stakeholders in which they expressed moral concerns regarding, for example, the question of whether EUCS will lead to a lower level of care for high-risk populations, or reinforce disability-based stigmatisation [15]. This was also described in a US focus group study by Cho et al. [18].

In the Netherlands, carrier screening is neither routinely offered, nor embedded in mainstream healthcare [19, 20]. In 2007, the Health Council of the Netherlands recommended studying both an offer of carrier screening for CF and HbPs in a large pilot, and the structural implementation of preconception care [21]. However, up till now, these recommendations have not been followed up, and carrier screening is only offered as local initiatives. In a Dutch founder population, screening for four AR disorders is available [8], a panel for nine disorders for the AJ community has been developed in two clinical genetic centres [22], and a few midwifery practices routinely offer ancestry-based prenatal HbP carrier screening for women at high risk [23]. Furthermore, despite the recognised importance of preconception care in general, this is only offered on a small scale, and not in a uniform manner [24]. In light of this context and the technological advances triggering the current transition in carrier screening, this paper aims to identify general and population-specific barriers and needs reflected by Dutch stakeholders regarding the implementation of (expanded universal) carrier screening.

## Methods

A qualitative study design was used and semi-structured interviews were held with key Dutch stakeholders in the practical and scientific field of carrier screening between December 2014 and September 2015. This qualitative research method was used to capture the range and diversity of perceived barriers and needs among different stakeholders. The Medical Ethical Committee of VU University Medical Center Amsterdam approved the study protocol.

### Theoretical framework

To describe the transition in carrier screening, and to structure the stakeholders’ views related to carrier screening, the so-called “constellation perspective” as described by Van Raak (2010) [25] was used. In this constellation perspective, there is a continuous interaction between three main elements of a larger societal system: the culture, the structure and the practice. Here, *culture* has been defined as “the set of shared values, perceptions, and interpretative frames of the involved actors” (e.g. perceptions regarding carrier screening), *structure* referred to how things are physically, financially, and institutionally organised, and *practice* involved the actual actions undertaken by the actors (individuals or groups) within the constellation (e.g. the interaction between patient and professional) [25]. Culture and structure are both structuring elements, and are shaped by the common practices of actors. At the same time, the actors’ actions are also influenced by the culture and structure of the constellation [25].

In general, the dominant constellation in medicine is often determined by a group of actors that are familiar with working according to a certain culture (thinking), structure (organising), and practice (doing). However, internal and/or external influences may cause this dominant constellation to be susceptible for change. These driving forces of change can originate from dynamics in organisation, demand, acceptability and/or technology [26]. Especially recent technical developments on EUCS make the current situation in carrier testing in healthcare susceptible to moving beyond ancestry-based screening.

### Recruitment and participants

**Fig. 1:**
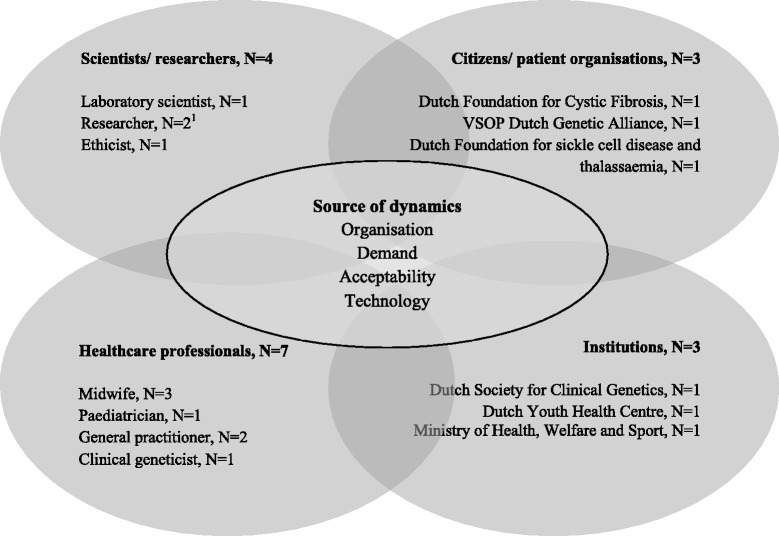
Interviewees categorised by the four different stakeholder groups in the Network of Actors model (adapted from [26]). ^1^One stakeholder is a researcher and a midwife, but is only assigned to the scientists/researchers group

A Network of Actors model [11, 26] was used to identify relevant stakeholder groups: scientists, including laboratory scientists, developing tests, healthcare professionals offering tests, citizens that might have a demand for carrier screening (e.g. patient organisations), and institutions developing policy and regulations (e.g. policy makers) (Fig. 1). Perspectives of the public are described elsewhere [20].

Stakeholders were non-randomly recruited from all four groups in the Network of Actors model [11], using role-based purposeful sampling. Stakeholders were selected based on their previous experience with carrier screening or because others identified them as having an important viewpoint on this topic. This sampling strategy resulted in twenty-five stakeholders who were approached for participation. Stakeholders were informed about the study and invited to participate via email. Of the people approached, three refrained as they were no longer involved in the topic or could not represent the organisation they worked for, and one interview was cancelled. Four people did not respond to the initial invitation and reminders. In total, seventeen interviews were conducted, after which data saturation was reached, and recruitment and data collection stopped. Five interviews were performed by two interviewers (EV/SvdH and KH) and the other twelve by a single interviewer (EV or KH). Interviews were conducted face-to-face, by telephone or via Skype. Informed consent was signed by all participants before the start of the interviews, and the conversations were audio recorded. Within two weeks, all interviewees received a summary of the interview for member checking.

### Interview guide

A semi-structured interview guide was developed by means of the literature, and the constellation perspective where its three main elements (culture, structure and practice) served as a basis for the formulation of interview questions. The guide covered the following topics: stakeholders’ previous experience with (offering) carrier screening, opinions on offering carrier screening, both ancestry-based and EUCS, opinions about (own) responsibility regarding the implementation of carrier screening and collaboration, views on the embedding of carrier screening in healthcare, and thoughts about commercial offers of carrier screening (see Additional file 1 for the interview guide). The results focussing on the moral considerations and the proposed benefits of EUCS are presented elsewhere [15]. During each interview, the questions were slightly adapted to the stakeholders’ specific knowledge and role.

### Data preparation and analysis

All interviews were typed out verbatim and thematic content analysis was performed. Coding started with reading all transcripts in detail, and labelling all recurring topics. All labels were ranked and clustered into main and subtopics based on the three main elements, i.e. culture, structure and practice, of the theoretical framework in order to identify important themes. Interviews were coded by three researchers independently (KH, EV and LH), and differences in coding were discussed until consensus was reached. Representative quotes were translated into English and are presented to illustrate the findings.

## Results

The interviews with key stakeholders revealed barriers and needs regarding the implementation of carrier screening that were clustered into themes on the three different levels of the constellation perspective (Table 1).

**Table 1:** Needs expressed by stakeholders regarding the implementation of carrier screening, related to culture, structure, practice

	Theme	Needs	Illustrative quotes
Culture	Desirability	Clarification about the, “who, and what” of screening • Who • What	*“Well, I think it should be available for people belonging to a risk population like for example those Afro-Caribbean people who have a chance of 1-in-7 to be a carrier of sickle cell disease.” (#1, healthcare professional)* *“I think [criteria to include disorders] is a trade-off that you should determine together, and by ‘together’, I’m afraid, I mean more people than just the geneticists.” (#13, healthcare professional)*
		Learning from existing niches • Insight in the demand for screening • Demand	*“I think it’s necessary to take some initiative [regarding screening]. […] Those small initiatives very often have a huge impact on the availability of such test offers.” (#5, scientist/researcher)* *“Look, it’s not a demand coming from the population itself, but it is all initiated by professionals emphasising freedom of choice. […] If there is some sense of urgency among the public, yeah that changes the situation.”(#16, healthcare professional)*
	Prioritisation	Different mind-set regarding the role of carrier screening • ‘Lobby’ to put carrier screening on the (political/professional) agenda, by: • Patient organisations • Healthcare professionals • Public	*“Well, and patient organisations need to be more active here. Some sort of lobby to put carrier screening on the political agenda.” (#6, healthcare professional)* *“So if we indeed reason from the field’s point of view, I think the first step will then be: talking to midwives via the branch organisation in order to get it on the agenda.” (#7, scientist/researcher)* *“But the Minister will decide whether it will happen or not. And who can address that? Well, that could be the women themselves, as that was also partly the case with prenatal screening.” (#15, healthcare professional)*
Structure	Infrastructure	Organising preconception care	*“First you should have some kind of [preconception care] facility before launching a website and newspaper articles, and God knows what. People should be able to go somewhere. That should be arranged first.” (#6, healthcare professional)*
		Organising an offer by several providers at different moments in time	*“I can also imagine that it’s not necessarily offered by only one person. If someone visits the general practitioner, he/she can do it, if someone visits the midwife, then he/she can do it as well. […] I would also be OK with the youth healthcare physicians offering it. If we can embed carrier screening there and they can counsel mothers while visiting the child health centre, that’s fine with me!” (#2, scientist/researcher)* *“I see a role for the youth healthcare physicians there [interconception care]. […] You can ask about the needs or the thoughts about having more children […] and you can inform about the possibilities of having tests.” (#11, representative of a Dutch institution)*
	Guidelines	Adequate and accessible guidelines	*“I think that the guidelines should include information about who is doing what (providing information), and what will happen when someone is found to be a carrier. I think that really should be clarified.” (#10, scientist/researcher)*
	Financial structures	Clarification about reimbursement and costs • Set a financial compensation for health professionals • Ensuring equity of access	*“Preconception care: that’s not working properly. […] There is no financial compensation. […]. We do have a preconception care guideline for general practitioners, […] but if you want that to work, you must first ensure that there is a rate, otherwise, you cannot implement it.” (#16, healthcare professional)* *“Autonomy is only possible if people have a choice. As soon as you say: ‘For 500 euros, you can have screening’, you already raise a barrier. So that should be something that is paid for from public funds, like the heel prick.” (#6, healthcare professional)*
	Counselling	Practical tools for professionals	*“It would be very helpful to have some kind of a practical tool or, if necessary, a website with concise and reliable information.” (#1, healthcare professional)*
		Focus on non-directive counselling	*“Considering the frameworks of non-directively offering carrier screening, parents-to-be should at least be informed about it [carrier screening], and should be asked whether they want to receive more information about the possibilities. […] This kind of information is different from informing someone about a healthy eating behaviour, quitting smoking and drinking when considering having children.” (#4, representative of a patient organisation)*
	Training and education	Organising training for professionals	*“They [professionals offering carrier screening] should be educated. […] It’s very complex, genetics. That’s something you should master in order to be able to provide information correctly.” (#11,representative of a Dutch institution)*
		Developing accessible information for the public	*“It’s really important to develop proper information for the public, and well, you should also take into account illiteracy.” (#13, healthcare professional)*
Practice	Demand for carrier testing	Individual request in daily practice	*“When people ask for it [carrier testing], I would certainly want to offer it, we should do it then. It doesn’t depend on me whether we are going to offer it or not.” (#1, healthcare professional)*
		Knowledge on consequences of knowing carrier status	*“I wonder if we know enough about the consequences of knowing.[…] Look, we know too little about the effects of carrier screening, and how parents feel about it afterwards. So yeah, I would recommend doing research on that”. (#11, representative of a Dutch institution)*
	Responsibility	Division of responsibility/ownership	*“Well, at this moment, no one is responsible, or at least, no one has taken that responsibility.” (#4, representative of a patient organisation)* *“It [the responsibility] should be left with the field, but you do need someone taking the lead.” (#8, representative of a patient organisation)*

### Culture: changing the way of thinking

The two main themes identified as important for changing culture regarding genetic carrier screening were desirability and prioritisation.

#### Desirability

Generally, respondents were positive about offering carrier screening, but the desirability was also debated and seemed to depend on two main considerations: the “who” — who should be offered screening: high-risk groups (ancestry-based) vs. population-wide — and the “what” — what disorders should be tested for. Carrier screening was considered more obvious for high-risk groups suffering from specific disorders, e.g. screening in populations of African ancestry or in founder populations. Reasons for preferring this were that these populations are at increased risk of being a carrier of several disorders, and that there was a degree of recognisability of the specific disorders for the high-risk groups. Disadvantages mentioned were an increasingly multi-ethnic society, concerns about the registration of ethnicity, and the risk of stigmatisation. Some therefore argued that screening should be offered more universal, as this would reduce the risk of stigmatisation. They, however, also indicated that such a population-wide offer would be too expensive and could lead to ethical dilemmas such as medicalisation of pregnancy and eugenics.

*“In my opinion, it should not be offered to everyone, as you will then medicalise the pregnancy”. (#12, healthcare professional)*

Regarding the inclusion of disorders in screening, stakeholders felt that there was a broad range of criteria that could be used, e.g. severity, prevalence and lethality of the disorder, the impact on quality of life, and the burden for parents. Many stakeholders therefore expressed a need for setting clear criteria that disorders have to meet in order to be included in a panel by a multidisciplinary team including all key actors (citizens, scientists, health professionals, and institutions), or by the Dutch Health Council. Focussing on the implementation of EUCS more specifically, stakeholders indicated that much can be learned from existing initiatives or niches on carrier screening, both ancestry-based and EUCS. One of the specific points to consider was the absence of an active demand by the public, apart from specific high-risk groups, for such an offer. According to the stakeholders, experiences from the current niches should clarify whether there is an actual demand for screening and more specifically what kind of offer (ancestry-based vs. EUCS) is preferred.

#### Prioritisation

A more generic theme arising from the interviews was the lack of priority for genetics in general in mainstream healthcare. Healthcare professionals indicated that offering carrier screening has no priority in daily practice, as the emphasis is mainly on curing patients instead of public health offers to healthy people (prevention). Moreover, they argued that when attention is paid to preventive aspects, professionals especially focus on traditional public health interventions targeting the well-known risk factors such as smoking and drinking during pregnancy, instead of on discussing genetic risks as genetic disorders were seen as rare.

*“You know, we still have to put everything in perspective. […] And, I think that quitting smoking has a bigger impact on someone’s health and unborn children at this moment.”(#13, healthcare professional)*

Additionally, stakeholders acknowledged the influence of the government and its changing policy regarding public health offers and preventive measures to healthy people. One of the stakeholders illustrated this by referring to the report *Preconception care: a good beginning* published by the Health Council of the Netherlands in 2007 [21], stressing the importance of preconception care.

*“And at that time, the former minister was willing to spend an amount of money on that, so that was good. But the next minister reversed that, so yeah, up till now no progress has been made.” (#6 healthcare professional)*

In case of structurally implementing carrier screening, stakeholders argued that a different mind-set towards preparing for a pregnancy, and the possible role of genetics and carrier screening is needed. Perceptions about how to achieve this culture shift differed. While some stakeholders acknowledged a role for patient organisations, others pointed out that healthcare professionals and professional organisations should take the lead. Additionally, the parallel was drawn with prenatal screening in the Netherlands, as pregnant women took a role in agenda setting themselves by asking for prenatal screening tests. This could also be the case for carrier screening (Table 1).

### Structure: changing the way of organising

Regarding structure, five themes emerged from the interviews: infrastructure; guidelines; financial structures; challenges during counselling; and training and education.

#### Infrastructure

Stakeholders perceived the lack of infrastructure in (primary) healthcare as an important barrier for implementing carrier screening, and discussed whether screening should be offered as a single screening programme or embedded in preconception care. According to respondents, when considering the structural implementation of preconception carrier screening, a well-organised infrastructure for preconception care in general, and guidelines are needed. Though preconception care was seen as a possibility, many stakeholders also raised concerns, e.g. the lack of knowledge among health professionals and the target population, and the lack of funding. Stakeholders furthermore argued that carrier screening should not necessarily be offered by one profession only. A proposed alternative was a structure where genetic carrier screening is offered by multiple providers: general practitioners, midwives, youth healthcare physicians, gynaecologists, clinical geneticists, and genetic counsellors. Though other providers were mentioned as well, most stakeholders agreed that screening should be offered by a medically trained professional to assure sufficient counselling. In light of the different potential providers, stakeholders also suggested different moments in time for offering carrier screening. Although offering screening preconceptionally was preferred, they also indicated that screening could be offered prenatally, for example embedded in existing routinely performed blood tests during pregnancy or between pregnancies (interconception care).

When discussing the desirability of a commercial offer, some stakeholders perceived the developments in commerce as inevitable, but many also expressed concerns, for example: doubts about the quality of the test, increasing inequality due to high costs, and creating a false sense of security.

*“I don’t know how reliable these companies are. When conducting a test outside university hospitals, who are the experts looking at the mutations then? Or is it just a fancy programme? It can all be automated. I think that’s a creepy development.” (#7, scientist/researcher)*

#### Guidelines

Stakeholders also expressed concerns regarding a lack of guidelines, complicating the offer of carrier screening or even informing people about it. Most respondents did acknowledge that some information was available, but it was often not in the right place, making it difficult to find or use. Stakeholders therefore outlined the need for adequate and accessible guidelines concerning indications for screening and issues that need to be addressed during pre- and post-test counselling. The development and adjustment of guidelines, however, was considered to be complex. Obstacles mentioned by stakeholders were the lack of financial resources, lack of time, and the many parties involved in designing guidelines.

#### Financial structures

Much uncertainty about the exact costs of carrier screening was expressed by the stakeholders, and in cases where the prices were known, they were perceived as high and unacceptable.

*“One of the arguments is that it is unethical, because the costs of those tests, you know, that is questionable, and a CF test is of course very expensive. Look, HbP that’s a tenner, but a CF test is quite pricey.” (# 2, scientist/researcher)*

This discussion seemed to be especially crucial when considering the implementation of EUCS. Despite the expectation that costs of carrier screening will decrease due to current technological developments, costs of testing and counselling remain substantial. Stakeholders argued that given the autonomy of possible users, attention should be paid to ensure equity of access. According to them, excessively high costs of screening might exclude people with a lower socio-economic status who might, for example, be more prone to HbPs in the Netherlands and would benefit most from screening. The autonomy principle is then violated. However, one respondent argued that it would be good to have people pay a small amount for screening as she thought that people would then make a more considered decision. Furthermore, it was mentioned that when discussing the embedding of carrier screening within preconception care, uncertainty about the reimbursement of such a consultation should be clarified first.

#### Challenges during counselling

Stakeholders expressed two challenges encountered during counselling for carrier screening. They acknowledged that people should receive sufficient information in order to make an informed decision about having screening or not. However, the complexity of the subject makes it difficult for healthcare professionals to provide this information. As counselling for ancestry-based screening for one or several disorders was already perceived as something complicated, stakeholders expected even more difficulties when offering EUCS.

*“You will have to explain the disorders properly. You have to explain life expectancy, and what it means to live with such a disorder. You will certainly be asked: can you cure this? You have to explain that very well. Is it treatable when we detect it in an early stage, can we cure it? […] And when it is a broad range of disorders, well you can imagine that it is too much information.” (#15, healthcare professional)*

Another stakeholder drew the parallel with the expansion of neonatal screening, from one to seventeen disorders over the years in the Netherlands, and argued that information could be provided in a layered fashion. Healthcare professionals can provide general information during counselling and refer to information leaflets or the internet for detailed information. Information can also be provided by ambassadors from their home country in their mother tongue. Furthermore, stakeholders argued that counselling for carrier screening requires a paradigm shift from directive advices to non-directive counselling. In order to tackle this, they expressed a need for practical tools (e.g. practice instructions – schematic overview of points to be discussed) that deal with the actual knowledge, and how to interpret lab results, but also include specific examples of how to counsel in a non-directive way.

#### Training and education

Stakeholders believed that sufficient knowledge about genetics and carrier screening is essential but also indicated that this important aspect is often lacking. Professionals are, however, not the only ones struggling with a lack of awareness and knowledge. Stakeholders indicated that the general public’s knowledge is also insufficient:

*“A bottleneck is, for example, the fact that men think they could never be a carrier when a baby is diagnosed with sickle cell disease because a baby has spent nine months in the mother’s womb, so it is always transferred by the mother”. (#3, healthcare professional)*

According to the stakeholders, the establishment of educational structures for both professionals and citizens are key in the implementation of carrier screening. They felt that more attention should be paid to genetics education in general, and carrier screening more specifically, by assigning a central role to this topic in the curricula of medicine students, and in postgraduate education for health professionals. Regarding informing the public, stakeholders argued that information can be provided by means of a media campaign or incorporating information in secondary education. As information should be accessible for everyone, illiteracy should also be taken into account.

### Practice: changing the way of doing

Two main practice-related themes emerged from the interviews as being of importance in the implementation of genetic carrier screening: demand for carrier screening and responsibility.

#### Demand for carrier screening

Stakeholders pointed out that the public can also influence implementation. When carrier screening is actually frequently requested in daily practice, healthcare professionals are most likely to be more willing to offer it. However, stakeholders argued that when it comes to preconception carrier screening, talking about a pregnancy wish might be perceived as taboo, and actively requesting and discussing information with healthcare professionals before conceiving is not yet self-evident.

*“It has also to do with the fact that it’s not self-evident that people will actively look for information themselves before starting a family.” (#4, representative of a patient organisation)”*

At the same time, stakeholders questioned whether individuals actually do something with the information about being a carrier; what are the consequences of knowing? Multiple stakeholders felt this should be studied further.

#### Responsibility

Responsibility was considered important and expressed as a clear need when discussing the implementation of carrier screening. However, according to stakeholders this is lacking at the moment. They argued that the government delegates the responsibility for the implementation of carrier screening to the healthcare professionals, whereas professionals generally expect the government to step in and take the responsibility to organise the debate about screening and to develop policy. However, an important nuance raised by one of the stakeholders is that placing the responsibility of execution with the government may also be misinterpreted. The neonatal screening and the vaccination programme were used as an illustration as although participation in these programmes is voluntary, a high uptake is pursued. Since a high uptake is not the primary aim when offering carrier screening, and mixed messages are to be avoided, stakeholders thought that the responsibility could also be assigned to an independent non-commercial organisation instead of the government.

## Discussion

This study identified different barriers and needs regarding the implementation of genetic carrier screening in a changing landscape by interviewing key stakeholders. They argued that, apart from requests in specific high-risk populations, there is still no actual demand for ancestry-based nor expanded universal carrier screening (EUCS) from the general population in daily practice, and a lack of priority for genetics and public health offers to healthy people planning to have children in mainstream healthcare. If an offer of genetic carrier screening were found to be desirable, stakeholders expressed a need for structural changes: a need for an infrastructure in healthcare for implementing carrier screening, a need for guidelines, financial and educational structures, and practical tools for counselling. Moreover, they felt a strong need for a clear division of responsibilities.

Since the current screening offers of EUCS are mainly technology-driven, it is important to attune to the actual demand [20]. After all, in the absence of demand, should we then continue offering? On the other hand, an offer can possibly generate demand as many people are not familiar with carrier screening yet, and can thus initiate an innovation curve. Previously, we have shown that the demand for screening may be influenced by awareness of and familiarity with genetic diseases and carrier screening [20]. While it has been acknowledged that in specific high-risk groups there is an actual demand for ancestry-based carrier screening [3, 7, 8], this is not yet evident for EUCS. However, a public health initiative for a population-wide screening offer is often not a reaction to individual requests, but a response to a significant public health problem, based on the need of (groups of) parents or physicians of children with a severe genetic disorder. A classic example is new-born screening for PKU, the first population screening program for a rare disease in many countries. Especially this aspect might explain why stakeholders stress the importance of an offer of carrier screening despite the limited individual demand.

Most stakeholders found an offer of ancestry-based carrier screening to be desirable, as was also described in the literature [27–29]. The desirability and acceptance of EUCS were, however, less obvious. Stakeholders expressed both practical and moral dilemmas [15], as was also shown by others [30, 31]. One dilemma concerned determining which disorders and how many should be included in a screening panel, and stakeholders acknowledged the importance of clear inclusion criteria set by a multidisciplinary team. Based on healthcare professionals’ opinions Lazarin et al. proposed a classification of disease severity for the inclusion in EUCS [32]. Stakeholders in our study furthermore feared that EUCS might lead to the medicalisation of pregnancy and to eugenics. These particular dilemmas were also mentioned in a Swedish interview study among health professionals [33]. We, and others, have also described that rather than increasing equity of access, EUCS might hinder this when specific mutations for high-risk groups were not included in the screening panels [15, 18].

Stakeholders also stressed that within healthcare, the priority (i.e. allocating time and money) is given to curing patients instead of to public health offers to healthy people (prevention). In the Swedish interview study, stakeholders expressed concerns about the costs of expanded carrier screening and the effects of its implementation on the budgeting of other healthcare areas. They indicated that money could better be spent on more vital diseases such as cancer [33]. In the case of insufficient resources, it is necessary to determine which tests are most important to provide, bearing in mind that allocating healthcare resources fairly requires the use of ethically reflected criteria [34]. Claims based on health and intervention need, e.g. the a priori probability of having a disease, appear to serve as the strongest normative basis [34]. Accordingly, ancestry-based carrier screening may be considered to have a higher need than EUCS, but the reverse can be argued as well: a service offering a carrier test for 100+ diseases has a higher chance of recognising carrier status of any disease than a service just testing for one disease, thus potentially generating more value.

Regarding the need for the development of organisational structures and ancillary matters such as guidelines, stakeholders did mention the potential of preconception care, but also the practical challenges involved. Other studies also report the many barriers that implementation encounters, for example, time constraints faced by healthcare professionals, few women attending preconception care consultations, and costs [24, 35, 36]. In the absence of a solid structure, it was suggested that screening could be offered at different moments in time by several providers. One of the options mentioned was interconception care, i.e. care provided between pregnancies, and that a specific role could be assigned to youth healthcare physicians providing preventive child healthcare. The potential of interconception care to new mothers is increasingly being discussed in the literature, and it is stated that its unique position to reach women and its expertise in preventive healthcare are facilitating factors [37, 38]. However, a disadvantage could be that in some cases, parents have already been confronted with the birth of an affected child without being aware of their increased risk. Another option might be a commercial structure. Though some respondents expected this to be an inevitable development, many expressed concerns: a lack of pre- and post-test counselling, and doubts about the quality of the test. These concerns have also been reported elsewhere [1, 16, 33], and already in 2010 policy on advertising and the provision of predictive genetic tests by so-called direct-to-consumer companies has been developed [39]. Without a clear structure within healthcare, commercial companies could potentially play a role in offering carrier screening, but the policy recommendations also apply [1, 39]. Thus proper monitoring and evaluation is needed here too. However, specific attention should be paid to the possibility of contributing to socio-economic inequalities by offering screening commercially [40].

In discussing who should take the responsibility or initiative for implementing genetic carrier screening or even developing organisational structures for preconception care in general, stakeholders continuously refer to each other. Given the Dutch legislation [41], this is not surprising since both parties (i.e. healthcare providers and public health authorities) find support for their viewpoints. Prevention is in many respects legally a responsibility of municipalities, but the maintenance and improvement of a support structure are national responsibilities. The Dutch policy on prenatal screening could serve as an example here, as indeed support structures to guarantee quality are a national responsibility [42]. To move forward, it is crucial to effectuate a new division of responsibilities. This can, for example, be achieved by having governments adopt an active role in discussing the responsible introduction of expanded carrier screening, as was suggested in recommendations by the European Society of Human Genetics [1], and in an Australian review on current CF carrier screening practices and the progress towards establishing a universal programme [43]. Another possibility might be acknowledging key actors (also referred to as “change agents” [26]) who are able to attune different stakeholders, and strategically plan the implementation of carrier screening.

The barriers and needs expressed by stakeholders continuously interact. A so-called collective sense of urgency among all key stakeholders is crucial for implementation in general, but also for genetics in particular [26]: an offer of carrier screening should be considered desirable and there should be an actual demand. When the interviews were conducted, this collective sense of urgency was far from evident, especially regarding EUCS. In the absence of an adequate structure and uncertainties about responsibilities, since 2016, Dutch clinical genetic centres of two university hospitals have taken the responsibility, and initiated the development of two new initiatives of offering EUCS. One comprises an offer via general practitioners within a research setting [44], and the other involves offering EUCS, non-reimbursed, via the clinical genetics department of a university hospital in Amsterdam [45]. Evaluation of uptake and outcomes of these initiatives will give insight in whether there is a demand for such an offer. Furthermore, arguments for testing and experiences with EUCS could be studied further.

### Strengths and limitations

By using a qualitative design, a broad range of barriers and needs regarding the implementation of carrier screening in a changing landscape was revealed that would have been difficult to recognise in a quantitative study. The use of theoretical frameworks furthermore helped identifying involved actors, and guiding and structuring the interpretation of the findings. In order to recruit the stakeholders, a role-based purposeful sampling strategy was used. Stakeholder were selected based on their previous experiences with carrier screening to enhance a lively, in-depth discussion about the topic. However, this method also entails disadvantages, such as, selection bias or an incomplete sample. To reduce the risk of bias, member checking, and researcher triangulation were used [46]. Despite the use of a model for identifying stakeholders, some perspectives are lacking in this study as for example gynaecologists did not respond to our invitation, as well as some professional organisations. Further, the perspective of the public itself or the citizens was not included, but has been described elsewhere [20]. Issues that were absent in our results but that were raised elsewhere, must be included in future quantitative studies, including specific barriers and needs. Lastly, as this study has been conducted in a very specific healthcare context, i.e. in the absence of a routine offer of carrier screening in mainstream healthcare, results cannot be directly translated to other countries.

## Conclusions

The absence of a collective sense of urgency for carrier screening, a lack of organisational structures, and uncertainty or even disagreement concerning the division of responsibilities for implementing and offering screening seem to be significant barriers to the implementation of genetic carrier screening. Stakeholders therefore suggest that change agents should be formally acknowledged to strategically plan broadening of current initiatives and attune different stakeholders. By using a theoretical framework for the identification and clustering of stakeholder perspectives regarding the implementation of carrier screening, this study provides a clear overview of relevant and urgent barriers and needs on different levels (culture, structure, and practice). These findings allow involved stakeholders to adequately address most important issues when implementing carrier screening in a changing landscape.

## Additional file

Additional file 1: Title of data: Semi-structured interview guide. This additional file includes the complete interview guide containing: an introduction to the interview study, the interview questions, and the closure of the interview. (DOCX 22 kb)

## Abbreviations

AJ: Ashkenazi Jewish
AR: Autosomal recessive
CF: Cystic fibrosis
EUCS: Expanded universal carrier screening
HbP: Haemoglobinopathy
PKU: Phenylketonuria
US: United States
