# Supplementary material for: Stakeholder perspectives on the implementation of genetic carrier screening in a changing landscape
Source: BMC Health Serv Res. 2017 Feb 16;17:146. doi: 10.1186/s12913-017-2083-9 (PMC5314610; doi:10.1186/s12913-017-2083-9)
Supplement: Additional file 1: — Title of data: Semi-structured interview guide. This additional file includes the complete interview guide containing: an introduction to the interview study, the interview questions, and the closure of the interview. (DOCX 22 kb) [file 12913_2017_2083_MOESM1_ESM.docx]

**Semi-structured interview guide**

**[Introduction]**

Good morning/afternoon/evening, thank you for making time for this interview. I am [*name*].

The current project aims to provide insight into the best approach for implementing (preconception) carrier screening in the Netherlands. During this interview, we are interested in what you consider to be the enabling and constraining factors for the implementation of carrier screening.

The interview will take approximately 45 minutes. All data will be anonymized. I would like to ask your permission to audio record this interview in order to process and analyse it properly. Afterwards, I will make a summary of the interview, which will be sent to you for verification.

Do you have any questions at the moment? Then I would like to ask you to sign this informed consent form. Signing this form indicates that you have understood the purpose of this study and the interview, and the process of analysing and reporting the data.

[*Signing of the informed consent form*]

I would like to start with the interview now.

[*Start audio recording*]

**[General/overarching questions]**

1. **Could you please explain in what way you are involved in carrier screening?**
2. **What is your role/task regarding (preconception) carrier screening?**
   1. Do you consider this an easy role/task for you to perform? Why/Why not?
3. **What is needed to implement and offer carrier screening?**
4. **What should be studied further before carrier screening can be implemented or not?** [*Asked at the end of the interview*]

**[Questions following the constellation perspective]**

| ***Elements of the constellation perspective*** | ***Questions*** |
| --- | --- |
| **Practice** | 1. **Who is, or should be, responsible for the implementation of** **(preconception) carrier screening? Why?** |
| **Culture** | 1. **Do you think carrier screening should be offered? Why/Why not?**     1. Under what conditions? 2. **To whom do you think carrier screening should be offered? Why?**    1. At what moment in a person’s life?    2. To specific subgroups only or to the entire population [*ancestry-based or expanded-universal carrier screening*]? 3. **What types of disorders should be included in (a) screening (panel)? Why?**    1. Who should determine this? |
| **Structure** | 1. **Do you collaborate with other people involved in (preconception) carrier screening?**     1. With whom?    2. Can you explain how you collaborate with others?    3. How would you describe the quality of this collaboration? 2. **What do you need in order to fulfil your specific role regarding (preconception) carrier screening?**     1. Do you feel that you have enough resources to fulfil this role? Why/Why not? 3. **In what way should carrier screening be embedded in healthcare?**     1. Where? What is needed to achieve this? 4. **Carrier screening is increasingly being offered by commercial companies. What do you think about this?** |

[**Closure**]

- Is there anything I forgot to ask that you think should be discussed?
- Do you have any questions?

Thank you for your time and your input.

[*Turn audio recorder off*]
